# Systematic Screening of Host Interactors for *Soybean mosaic virus* Proteins Identifies Four Soybean (*Glycine max*) Antiviral Factors

**DOI:** 10.3390/plants15111650

**Published:** 2026-05-27

**Authors:** Niu Niu, Wenxia Li, Zikai Zhou, Hada Wuriyanghan

**Affiliations:** 1 School of Life Science, Inner Mongolia University, Hohhot 010070, China; niuniu1901@126.com (N.N.); liwenxia23@163.com (W.L.); 67983286@163.com (Z.Z.); 2 Key Laboratory of Herbage & Endemic Crop Biology, Ministry of Education (IMU), Hohhot 010070, China

**Keywords:** *Glycine max*, *Soybean mosaic virus*, virus–host interaction, antiviral factors, GmSiPPase

## Abstract

*Soybean mosaic virus* (SMV) establishes successful infection through the coordinated action of multiple viral proteins, yet how these components are collectively engaged in host processes remains unclear. Using 10 SMV-encoded proteins as baits, we screened a soybean (*Glycine max*) cDNA library using the yeast two-hybrid (Y2H) method and obtained 200 positive clones corresponding to 147 nonredundant candidate host proteins. Integrative analyses indicated that these interacting proteins were mainly associated with host processes related to photosynthesis and energy metabolism, as well as protein quality control (PQC), and that their promoters were enriched in light- and stress-responsive elements. Photosynthesis-related genes were more strongly perturbed in the susceptible cultivar, whereas PQC-module genes showed overall downregulation in both resistant and susceptible cultivars upon SMV infection. Y2H, Bimolecular Fluorescence Complementation (BiFC), and transient expression assays identified four soybean resistance factors that interact with SMV proteins. These factors delayed systemic spread and continued to restrict viral proliferation after systemic infection was established. Notably, SMV accumulation partially rebounded when viral proteins were co-expressed with GmSTOP1, GmHrBP1, or GmGIP2, while co-expression of GmSiPPase and NIa-Pro further inhibited viral proliferation. In summary, this study maps the host-targeting profile of SMV across multiple viral components and provides clues to the SMV–soybean interaction network and antiviral gene resources in soybean.

## 1. Introduction

*Soybean mosaic virus* (SMV) is one of the most destructive viral pathogens of soybean (*Glycine max*) worldwide, causing substantial economic losses [1]. Transmitted nonpersistently by aphids and vertically through seeds, SMV causes leaf mosaic, chlorosis, wrinkling, and seed coat mottling, resulting in severe quality deterioration, yield losses of up to 25–50%, and, in severe cases, complete crop failure [2]. Current studies on the genetic basis of SMV resistance have focused mainly on quantitative trait locus (QTL) mapping and the functional characterization of individual resistance genes [3,4]. However, there is still a gap in comprehensive understanding of how different viral components engage host factors in a coordinated manner during infection.

SMV, a member of the genus Potyvirus in the family Potyviridae, has a single-stranded positive-sense RNA genome of approximately 9.6–10 kb. The viral polyprotein is processed by virus-encoded proteases into at least 11 mature proteins, including protein 1 (P1), helper component-proteinase (HC-Pro), coat protein (CP), 6 kDa protein 1 (6K1), cylindrical inclusion protein (CI), 6 kDa protein 2 (6K2), nuclear inclusion a-viral protein genome-linked (NIa-VPg), nuclear inclusion a-protease (NIa-Pro), nuclear inclusion b (NIb), protein 3 (P3), and P3N-PIPO, which is generated by a frameshift within P3 [5,6]. Like other positive-sense RNA viruses, potyviruses have limited coding capacity and therefore rely on multifunctional proteins that interact extensively with host factors to support replication, movement, transmission, and immune evasion [7,8,9]. HC-Pro represents a typical example of such multifunctionality, as it contributes to RNA silencing suppression, aphid transmission, and systemic infection. Previous studies have shown that HC-Pro recruits AGO1 through its WG motif to participate in the stable production of viral particles and the efficient establishment of systemic infection. HC-Pro also associates with the host decapping factor VARICOSE (VCS), thereby contributing to suppression of antiviral RNA silencing and systemic infection [10,11,12]. In addition, the interaction of NIa-VPg with the host translation initiation factors eIF4E or eIFiso4E is critical for infection by multiple potyviruses [13]. 6K2 and NIb promote the assembly of viral replication complexes (VRCs) by recruiting host factors involved in vesicle trafficking and translational elongation [14,15]. P3N-PIPO and NIa-VPg promote viral movement and systemic spread by recruiting PCaP1 to plasmodesmata and counteracting the negative regulator REM1.2, respectively [16]. Furthermore, VPg/6K2, NIb, and NIa-Pro have been reported to suppress host immunity by antagonizing autophagy, interfering with salicylic acid (SA) signaling through NPR1 SUMOylation- and phosphorylation-mediated regulation, and promoting the degradation of host defense-related proteins [17,18,19].

In recent years, several studies on SMV–host interactions have provided initial insights into the molecular basis of SMV pathogenicity. Among SMV proteins, P3 is one of the best-characterized SMV proteins and has been shown to interact with the host translation elongation factor eEF1A, induce the unfolded protein response, and promote SMV accumulation [20]. Further studies of host factors such as GmRHP, GmGLU, and GmCYB5-4 have shown that P3 is involved in multiple host processes and can be associated with host factors that either promote or restrict viral infection. Among these factors, GmRHP, an endoplasmic reticulum membrane-associated protein, may promote viral infection by remodeling the virus-associated membrane system [21]. GmGLU is thought to promote SMV cell-to-cell movement by reducing callose deposition at plasmodesmata [22]. By contrast, GmCYB5-4 may contribute to antiviral responses through endoplasmic reticulum stress [23]. In addition to P3, other SMV proteins, including 6K1, NIa-Pro, and NIb, also target host factors to influence infection. The interaction between 40S ribosomal protein S8 (RPS8) and 6K1 has been shown to enhance soybean susceptibility to SMV [24], whereas the chloroplast-associated proteins PSaC and ATPsyn-α, which interact with NIa-Pro and NIb, respectively, are associated with the restriction of viral accumulation and systemic infection [25]. These findings indicate that different SMV proteins target diverse host pathways, including translational regulation, membrane trafficking, cell-to-cell movement, and energy metabolism. However, current knowledge is still based largely on studies of individual viral proteins and a limited number of host factors, and systematic comparative analyses across multiple viral components are still lacking.

Although studies of potyvirus–host interactions have increased in recent years, the depth of investigation remains highly uneven across different virus–host pathosystems [26]. By contrast, large-scale host interaction networks have been identified in model systems such as *Turnip mosaic virus* (TuMV) and *Arabidopsis thaliana*, but subsequent studies have shown that only a small subset of these factors play key roles in infection [27]. This suggests that identifying core host factors from complex physical interaction profiles requires evidence from multiple levels of analysis. At present, parallel screening, cross-component comparison, and systematic network analysis for multiple SMV proteins remain largely unexplored. Based on this rationale, we used 10 mature SMV proteins as baits (excluding P3N-PIPO) to systematically screen for interacting soybean proteins and combined multilevel analyses with functional validation to characterize the major features of the SMV–host interaction network. By functionally validating representative candidates from different modules, this study aims to provide a framework for understanding host-dependent mechanisms during SMV infection and identifying new antiviral gene resources.

## 2. Results

### 2.1. Identification of Host Interacting Proteins for Ten SMV Proteins

**Figure 1:**
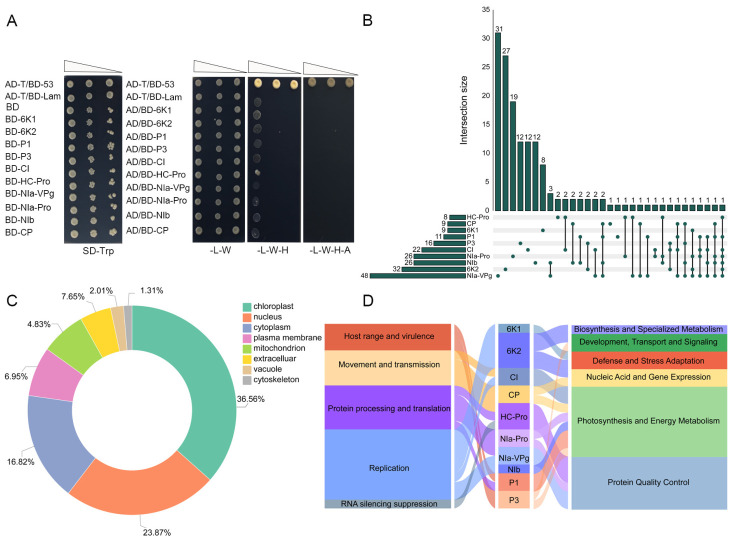
Screening of interacting proteins and functional overview of ten SMV-encoded proteins. (**A**) Autoactivation and toxicity tests of ten SMV-encoded proteins in yeast. Cotransformed Y2HGold cells were grown on SD/-Trp, DDO, TDO, and QDO media. AD-T/BD-p53 and AD-T/BD-lamin served as positive and negative controls, respectively. (**B**) Summary of host proteins identified from screening with the ten SMV-encoded proteins. The horizontal bars on the left indicate the total number of positive host protein entries recovered for each viral bait (200 in total). On the right, the vertical bars summarize the 147 nonredundant host proteins, and the connected dot matrix indicates proteins shared among different bait proteins. Single dots represent proteins identified only with one specific SMV protein. (**C**) Predicted subcellular localization of 147 candidate SMV-interacting proteins. Protein number and percentage were calculated according to WoLF PSORT prediction categories. (**D**) Functional association analysis of 147 candidate SMV-interacting proteins and ten SMV proteins.

To systematically identify soybean proteins that interact with SMV component proteins, we used 10 mature proteins encoded by the SMV genome (P1, P3, CI, HC-Pro, NIa-VPg, NIb, 6K1, 6K2, CP, and NIa-Pro) as baits to screen a soybean cDNA library using the yeast two-hybrid (Y2H) system. Before library screening, we tested the 10 bait proteins for autoactivation and toxicity in the yeast strain Y2HGold (Figure 1A). None of the baits showed detectable toxicity or self-activation, indicating that they were suitable for subsequent interactor screening. The screen yielded 200 positive interaction entries. After removing redundant hits derived from repeated capture of the same host protein by different viral baits, 147 nonredundant candidate interacting proteins were retained for further analysis (Figure 1B, Figures S1 and S2). Prediction of subcellular localization using WoLF PSORT showed that these 147 candidate proteins were mainly localized to the chloroplast (36.65%), followed by the nucleus (23.87%) and the cytoplasm (16.82%) (Figure 1C). Based on SoyBase functional annotation and manual curation, we classified these candidate proteins into six functional modules: biosynthesis and specialized metabolism, photosynthesis and energy metabolism, protein quality control (PQC), nucleic acids and gene expression, development, transport and signaling, and defense and stress adaptation. We then examined their associations with the known biological functions of SMV proteins (Figure 1D). Most SMV proteins were associated primarily with host factors related to photosynthesis and energy metabolism. In addition, the interacting proteins of HC-Pro, NIa-Pro, and NIa-VPg were also enriched in the PQC module. These results indicate that different SMV components not only exhibit certain differences in their host targets but also demonstrate distinct functional biases.

### 2.2. SMV-Interacting Proteins Cluster in Photosynthesis, Energy Metabolism and PQC

**Figure 2:**
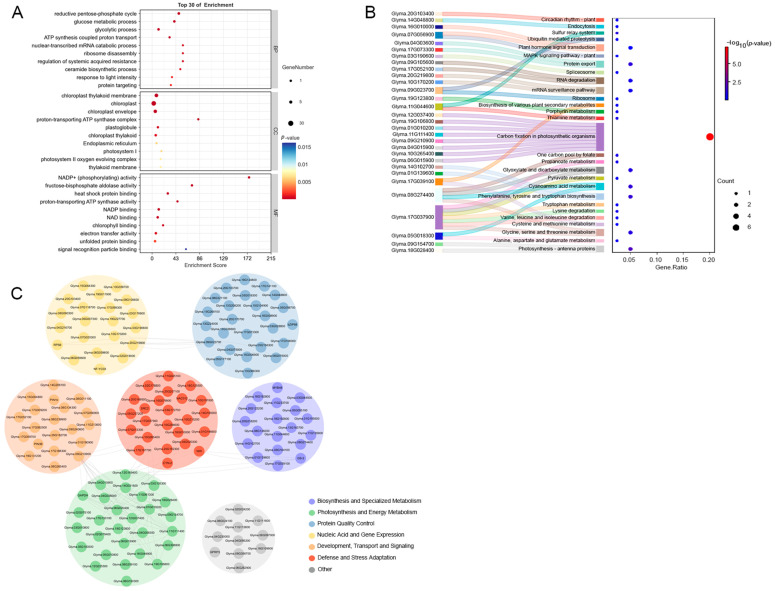
Enrichment features and modular associations of 147 candidate SMV-interacting proteins. (**A**) GO enrichment analysis of the 147 candidate proteins. The top 30 enriched GO terms are shown. Bubble size indicates protein number, and color indicates adjusted *p*-value. (**B**) KEGG pathway enrichment analysis of the 147 candidate proteins. The Sankey diagram shows protein–pathway relationships, and the bubble plot summarizes pathway significance. Bubble size indicates protein number, and color indicates adjusted *p*-value. (**C**) Modular association network of the 147 candidate proteins. Nodes are colored by functional category, and edges indicate STRING-predicted associations (confidence score ≥ 0.7). Proteins without predicted interactions were placed into their corresponding modules to provide a comprehensive overview of the functional distribution of all proteins. The panel is intended to illustrate the functional composition of the SMV interaction set, rather than to identify central nodes (hub nodes) based on network topology.

To further define the potential functions of the 147 candidate interacting proteins during SMV infection, we performed Gene Ontology (GO) and Kyoto Encyclopedia of Genes and Genomes (KEGG) enrichment analyses and constructed a module-based association network. GO enrichment analysis showed that a large proportion of candidate proteins were assigned to chloroplast-related cellular components, possessed molecular functions such as heat shock protein binding and unfolded protein binding, and were mainly involved in biological processes including the reductive pentose phosphate cycle and glucose metabolism (Figure 2A). KEGG analysis further supported this pattern, showing that the candidate proteins were significantly enriched in carbon fixation in photosynthetic organisms, followed by photosynthesis-antenna proteins, as well as in the pathways related to ubiquitin-mediated proteolysis and protein export (Figure 2B).

We then constructed a modular association network. This network was designed to illustrate the functional distribution of the 147 candidate proteins within the SMV interaction set, rather than to reconstruct a complete protein–protein interaction map. The photosynthesis and energy metabolism module and the PQC module contained the largest numbers of interacting proteins, with the former showing particularly dense predicted internal connections. Although other modules contained fewer interacting proteins, they still showed potential associations with the major modules through a small number of predicted links, suggesting possible cross-module coordination among host functional units (Figure 2C). Together, these results indicate that SMV-interacting proteins are mainly involved in host processes related to photosynthesis and energy metabolism, as well as protein quality control, while chloroplast-associated components and pathways are particularly prominent within the former.

### 2.3. Promoter Features and Expression Patterns of Candidate Host Genes

**Figure 3:**
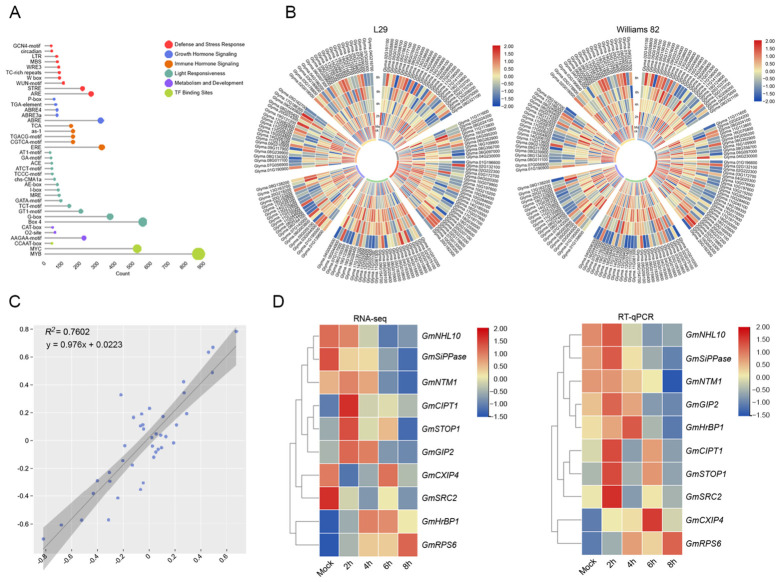
Promoter cis-element prediction and expression analysis of candidate host gene. (**A**) Prediction of promoter cis-acting elements in genes encoding the 147 candidate proteins. The lollipop plot shows the types and numbers of predicted cis-elements within the 2 kb region upstream of the translation start codon (ATG) of each candidate gene. (**B**) Circular heat maps showing the expression changes of 147 candidate host genes in the resistant soybean(*Glycine max*) cultivar L29 and the susceptible cultivar Williams 82 at 0, 2, 4, 6, and 8 hpi. Genes and functional categories (color scheme as in Figure 2C) are indicated on the outer ring, and colors represent log_2_ fold change. (**C**) Correlation analysis of RNA-seq and RT-qPCR results for 10 candidate genes. The scatter plot shows the linear regression relationship between RNA-seq values (*x*-axis) and RT-qPCR values (*y*-axis) for the selected candidate genes. (**D**) Comparison of expression patterns of the 10 selected genes between RNA-seq (left) and RT-qPCR (right). Data were row-scaled before visualization.

To characterize the regulatory features and early transcriptional responses of the candidate host genes, we first analyzed cis-acting elements in their promoter regions and then examined their expression dynamics during SMV infection. First, we retrieved upstream promoter sequences of these genes from soybean genomic database. Promoter analysis revealed broad enrichment of diverse cis-acting elements in the upstream regions of the 147 candidate genes. Among these elements, MYB and MYC sites, as key regulatory elements associated with secondary metabolism, development, and jasmonate signaling, were the most abundantly represented. They were followed by the light-responsive elements BOX4 and G-box, as well as the hormone-responsive elements ERE and ABRE (Figure 3A), suggesting that the candidate genes may be transcriptionally associated with light responses, hormone signaling, and stress responses.

We next examined the early transcriptional responses of these candidate genes during infection by generating circular heatmaps for the resistant soybean cultivar L29 and the susceptible cultivar Williams 82 at mock, 2, 4, 6, and 8 h post inoculation (hpi) (Figure 3B). Overall, the genes from different functional modules showed some variation in early transcriptional responses between the resistant and susceptible cultivars. Within the defense and stress adaptation module, a subset of genes displayed relatively high basal expression in L29 before inoculation and showed stronger induction at 2–4 hpi, whereas the corresponding genes in Williams 82 showed weaker and less sustained induction. This pattern suggests that immune-related responses were activated more rapidly in the resistant cultivar background. The photosynthesis and energy metabolism module showed different trends in the two cultivars. In L29, the proportions of upregulated and downregulated genes were similar, whereas more genes in Williams 82 exhibited transcriptional repression, indicating that photosynthesis-related processes were more strongly affected in the susceptible cultivar background. In the PQC module, genes in both cultivars showed an overall downward trend, but the decrease was generally more pronounced in L29, indicating that PQC-related genes differed in the strength of their transcriptional response to SMV infection between resistant and susceptible backgrounds.

We then selected 10 candidate host factors for experimental validation based on their primary functional annotations and literature-supported relevance to host responses during infection, while retaining several functional backgrounds among the selected targets (Table S3). Their expression changes after SMV infection in Williams 82 were first examined by RT-qPCR. Correlation analysis showed an overall agreement between the RT-qPCR and RNA-seq datasets (*R*^2^ = 0.7602), indicating that the RNA-seq data reliably reflected the transcriptional responses of these candidate genes during infection (Figure 3C). Consistently, paired heatmaps comparing the two datasets showed that although the magnitude of expression change and clustering order differed to some extent, the overall expression trends were similar for most genes (Figure 3D).

### 2.4. Functional Validation and Antiviral Activity of Ten Soybean Factors

**Figure 4:**
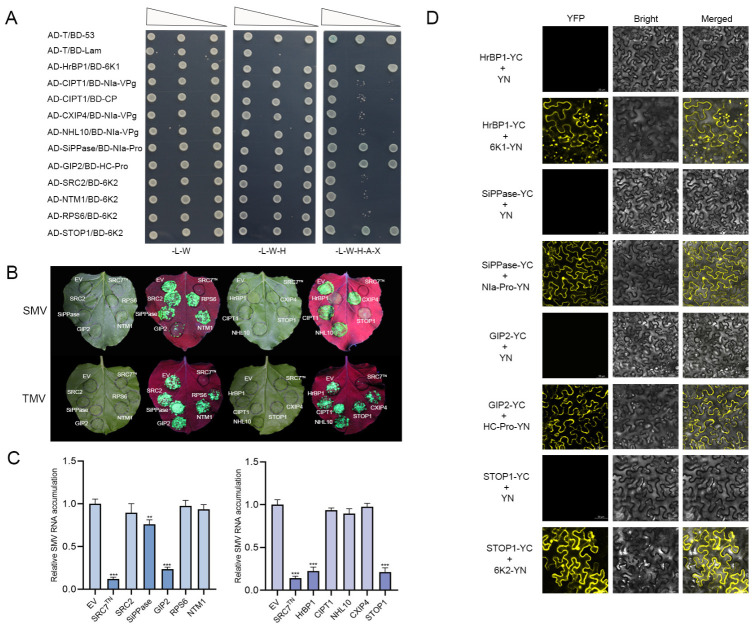
Identification of antiviral host factors and their interactions with corresponding SMV proteins. (**A**) Y2H revalidation of interactions between 10 candidate proteins and their corresponding SMV proteins. Yeast cells carrying the indicated constructs were grown on selective media. Serial dilutions of yeast cultures (10^−1^, 10^−2^, and 10^−3^) were used. AD-T/BD-p53 and AD-T/BD-lamin served as positive and negative controls, respectively. (**B**) Effects of candidate genes on SMV and TMV accumulation in the transient expression system of *Nicotiana benthamiana*. Candidate genes were co-expressed with SMV-GFP, and virus accumulation was observed under a handheld UV lamp (Wavelength = 365 nm) at 5 days post inoculation (dpi). Empty vector (EV) was used as the negative control, and the SRC7^TN^ truncation construct generated previously in our laboratory was used as the positive control. TMV-GFP was included as a parallel treatment for a preliminary comparison of the antiviral potential of the candidate genes. (**C**) RT-qPCR quantification of SMV RNA accumulation in infected leaves shown in panel B. Data represent the mean ± SD of three independent biological replicates. Asterisks indicate significant differences compared with the EV control (** *p* < 0.01, *** *p* < 0.001; one-way ANOVA followed by Dunnett’s multiple-comparison test). adjusted *p*-values are provided in Supplementary Tables S4 and S5. (**D**) BiFC validation of the interactions between four antiviral proteins and their corresponding SMV proteins in *N. benthamiana* cells. cYFP and nYFP fusion constructs were co-infiltrated into *N. benthamiana* leaves for transient expression. Empty nYFP fusion constructs were used as negative controls. At 48 hpi, YFP fluorescence was examined by confocal microscopy. EYFP signals were detected with excitation at 514 nm and emission at 520–550 nm. Scale bars in all panels = 50 μm.

We next re-examined the physical interactions between the 10 selected host factors and their corresponding SMV proteins by Y2H assay. All candidate factors interacted with their corresponding viral bait proteins. Among them, GmSTOP1 (sensitive to proton rhizotoxicity 1), GmHrBP1 (harpin receptor-binding protein 1), GmSiPPase (soluble inorganic pyrophosphatase), and GmGIP2 (glucanase-inhibiting protein 2) displayed stable interactions with 6K2, 6K1, NIa-Pro, and HC-Pro, respectively, even under more stringent selection conditions (Figure 4A).

We then evaluated the potential roles of these 10 candidate genes during SMV and *Tobacco mosaic virus* (TMV) infection using an *Agrobacterium*-mediated transient expression system in *Nicotiana benthamiana*. The organization of the GFP reporters in the SMV-GFP infectious clone and TMV-GFP (pJL24) vector is shown in Supplementary Figure S1. Among these 10 proteins, GmSTOP1, GmHrBP1, GmSiPPase, and GmGIP2 exhibited antiviral activity against SMV, whereas no corresponding antiviral phenotype was observed in the parallel TMV assay (Figure 4B). RT-qPCR analysis further confirmed that SMV RNA accumulation was significantly reduced in leaves expressing *GmSTOP1*, *GmHrBP1*, *GmSiPPase*, or *GmGIP2* compared with the EV control (Figure 4C), and fluorescence-based quantification showed a similar trend (Figure S2A). These results indicate that the four host factors inhibit SMV accumulation in the transient expression system. Given that these candidate factors were identified from the SMV interactor screen and that all subsequent validation experiments were carried out in the context of SMV components, the following analyses focused primarily on their effects on SMV.

Based on these results, we further validated the interactions between the four antiviral proteins and their corresponding viral proteins in *N. benthamiana* cells using bimolecular fluorescence complementation (BiFC) assays (Figure 4D and Figure S3). All four interactions were confirmed *in planta*, but each showed a distinct subcellular distribution pattern. The GmHrBP1–6K1 interaction signal was detected at the plasma membrane and chloroplasts; GmSiPPase interacted with NIa-Pro at the plasma membrane and in the nucleus; GmGIP2 interacted with HC-Pro at the plasma membrane; whereas the GmSTOP1–6K2 interaction signal was concentrated in the nucleus and at the plasma membrane.

### 2.5. Four Soybean Factors Restrain SMV Spread Both Locally and Systemically

Although the above four host factors showed clear antiviral effects against SMV, the spatial basis of their functions and their impacts on systemic viral spread remained unclear. In addition, whether the interacting viral proteins interfered with their antiviral activities differently was unknown. Therefore, we further examined their subcellular localization, effects on SMV systemic spread, and responses to co-expression with the interacting viral proteins.

**Figure 5:**
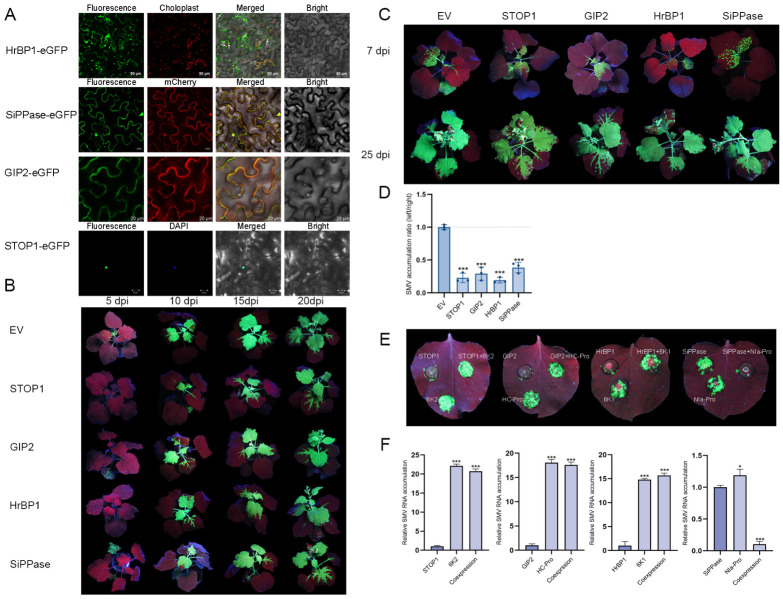
Subcellular localization and antiviral function verification of four resistance factors. (**A**) Subcellular localization of four antiviral proteins. *Agrobacterium* strains carrying recombinant pNC-Green-SubC constructs were infiltrated into *N. benthamiana* leaves, and the localization of each eGFP-tagged protein was examined by confocal microscopy at 48 hpi. DAPI staining or Histone3-mCherry transgenic *N. benthamiana* plants were used to visualize the nucleus, LTI6b-mCherry was used as a plasma membrane marker, and chlorophyll autofluorescence was detected at 637–720 nm. White arrowheads indicate representative chloroplast-associated punctate signals. (**B**) Effects of four antiviral genes on systemic spread of SMV. Each gene was co-expressed with SMV-GFP in basal leaves of *N. benthamiana*, and systemic infection was monitored under UV light at 5, 10, 15, and 20 dpi. EV served as the negative control. (**C**,**D**) Local inhibition assay after systemic infection had been established. By 7 dpi, when SMV-GFP had spread to the upper leaves, each host factor was transiently expressed on the left half of a newly infected upper leaf, while the right half served as the internal control. Viral accumulation on the two halves was compared at 25 dpi. In panel D, SMV RNA accumulation was quantified by RT-qPCR and calculated as the left/right ratio. For fluorescence-based quantification shown in Supplementary Figure S2B, GFP-positive areas were measured from the green channel using ImageJ (ij154-win-java8), normalized to the total area of the corresponding half-leaf region, and then expressed as the left/right ratio. (**E**,**F**) Effects of the corresponding viral proteins on the antiviral activities of the four host factors. For each interaction pair, the host factor alone, the viral protein alone, and their co-expression were compared in the same leaf, and all sectors were uniformly inoculated with SMV-GFP at 1 dpi. Local SMV accumulation was examined at 5 dpi. In panel F, SMV RNA accumulation was quantified by RT-qPCR. For the quantified data in panels (**D**,**F**), values represent the mean ± SD of three independent biological replicates. Asterisks indicate significant differences (* *p* < 0.05, *** *p* < 0.001) as assessed by one-way ANOVA followed by Dunnett’s multiple-comparison test, using EV in panel D and the host factor-alone group in panel F as the respective controls. Exact adjusted *p*-values are provided in Supplementary Tables S4 and S5.

Subcellular localization analysis revealed distinct spatial distribution patterns for the four antiviral proteins in *N. benthamiana* cells (Figure 5A). GmSTOP1-eGFP fluorescence was mainly enriched in the nucleus; GmGIP2-eGFP was localized to the plasma membrane; GmHrBP1-eGFP showed plasma membrane localization and punctate signals in chloroplast, whereas GmSiPPase-eGFP was distributed at both the plasma membrane and the nucleus.

We next examined the effects of these four antiviral genes on the systemic spread of SMV (Figure 5B). Each gene was co-expressed with SMV-GFP in basal leaves of *N. benthamiana*, and infection was monitored over time. By 10 dpi, the EV control had already developed clear systemic infection, with strong GFP signals observed in upper newly emerged leaves and along the veins. In contrast, the extent of SMV spread upon overexpression of the four host-factors was generally lower than that in the EV control, as reflected by fewer infected leaves or a reduced area of GFP signal distribution. By 15–20 dpi, although SMV eventually established systemic infection in all treatments, overall GFP signals still remained lower than in EV, suggesting that all four host factors reduced the apparent systemic accumulation of SMV-GFP.

A local inhibition assay further showed that these four antiviral proteins retained inhibitory activity even after systemic infection had been established (Figure 5C). By 7 dpi, when GFP signals were visible in upper tissues, each of the four genes was transiently expressed in the left half of a subapical leaf from systemically infected plants, whereas the right side of the same leaf was left untreated as an internal control. By 25 dpi, SMV had spread further and accumulated strongly on the control side, whereas its accumulation was clearly suppressed on the side expressing the candidate genes. RT-qPCR quantification of SMV RNA accumulation from the left and right halves of the same leaves further showed that the left/right ratios of all four host factor treatments were lower than that of the EV control (Figure 5D), confirming that these host factors can still restrict viral accumulation in infected leaves after systemic infection has been established.

Finally, we examined how the corresponding viral proteins affected the antiviral activities of these candidate proteins (Figure 5E). Expression of GmSTOP1, GmGIP2, or GmHrBP1 alone markedly suppressed local SMV accumulation. However, when each of these proteins was co-expressed with its interacting viral protein (6K2, HC-Pro, or 6K1, respectively), SMV accumulation in the same region was clearly restored relative to expression of the host protein alone. By contrast, when GmSiPPase was co-expressed with NIa-Pro, local SMV accumulation was decreased further instead of restoration. RT-qPCR quantification of SMV RNA accumulation further showed that the corresponding viral proteins affected the antiviral activities of the four host factors to different extents (Figure 5F). The corresponding fluorescence-based quantification showed a similar trend (Figure S2C).

## 3. Discussion

In this study, Y2H library screening identified 147 potential soybean interactors for 10 SMV-encoded proteins (excluding P3N-PIPO), and four of these factors were further shown to restrict SMV accumulation. Different viral proteins retained component-specific host targets while also sharing a subset of host factors. This interaction pattern suggests that SMV exploits the host factors in a coordinated rather than isolated manner, with different viral proteins targeting distinct host biological processes to collectively reshape a host environment that favors viral infection.

Among the six functional modules, the photosynthesis and energy metabolism module was particularly prominent, mainly reflected by the marked overrepresentation of chloroplast-associated components and metabolic pathways. Subcellular localization prediction and functional enrichment analyses further showed that SMV-interacting proteins were strongly enriched in chloroplast-related compartments and were mainly associated with primary metabolic processes, including the reductive pentose phosphate cycle and glucose metabolism. These findings suggest that viral interference is initially directed toward cellular compartments closely linked to energy supply and carbon metabolism. Chloroplasts have dual roles during potyvirus infection: they not only provide metabolic support and a membrane platform for viral replication and infection, but also produce defense-associated phytohormones, including salicylic acid (SA) [28]. Several soybean chloroplast proteins, such as GmPAP2.1, GmPSaC, and GmATPsyn-α, have been shown to participate in antiviral immunity through SA-dependent signaling or RNA silencing-related pathways [29,30]. Together with our observation that photosynthesis-related genes were more strongly repressed in the susceptible background and that light-responsive elements such as BOX4 and G-box were enriched in host promoters, these findings suggest that SMV–host interactions likely involve not only perturbation of chloroplast metabolism but also possible interference with chloroplast-associated defense functions.

Protein quality control (PQC) represents another major host process preferentially targeted by SMV. Previous studies have shown that viruses manipulate the host ubiquitin–proteasome system (UPS) to prevent degradation and promote intracellular conditions favorable for infection [31]. As an essential part of this module, molecular chaperones mediate protein folding, assembly, and turnover, and many heat shock proteins have been implicated in the movement and symptom development of different potyviruses [32]. In our dataset, the targets of HC-Pro, NIa-Pro, and NIa-VPg were enriched in this module, and the significant enrichment of terms such as “heat shock protein binding” and “ubiquitin-mediated proteolysis” further suggests that SMV may interfere with the host proteostasis network to support its own protein processing and infection cycle. Expression profiling showed that the PQC module was downregulated in both cultivars, but the decrease was more pronounced in L29 than in Williams 82, indicating that protein homeostasis-related processes respond differently in resistant and susceptible host backgrounds. Whether this difference reflects an active host defense that limits viral protein folding or a differential consequence of infection remains unclear and will require functional validation of key chaperones and proteasome-associated factors.

Preliminary functional assays showed that transient expression of GmSTOP1, GmHrBP1, GmSiPPase, and GmGIP2 locally suppressed SMV accumulation in tobacco leaves, delayed systemic spread, and continued to inhibit viral proliferation after systemic colonization was established. These results support the antiviral potential of the four proteins in a heterologous transient-expression system. In soybean, RT-qPCR analysis showed that the corresponding genes were significantly induced during the early stage of SMV infection (2–4 hpi), indicating that they are transcriptionally responsive to SMV infection in the native host. However, because the expression and functional assays were performed in different host systems and at different infection stages, whether this early transcriptional induction is directly linked to antiviral activity remains to be tested in soybean at matched infection stages. The co-expression assays further revealed that the interacting viral proteins affected these host restriction factors in different ways. SMV accumulation partially rebounded when GmSTOP1, GmHrBP1, or GmGIP2 was co-expressed with 6K2, 6K1, or HC-Pro, respectively, whereas SMV accumulation decreased further when GmSiPPase was co-expressed with NIa-Pro. This divergence suggests that the outcome of each host–virus interaction may depend on the host process in which the restriction factor operates.

At the membrane-associated host–virus interface, GmHrBP1 and GmGIP2 represent host restriction factors associated with the defense and stress adaptation module and the PQC module, respectively. GmHrBP1 interacted with 6K1 in plasma membrane- and chloroplast-associated compartments, and its antiviral effect was partially attenuated by 6K1. As a Harpin receptor-binding protein, HrBP1 can trigger systemic acquired resistance (SAR) and activate defense pathways involving SA, ethylene (ET), jasmonic acid (JA), and MAPK-related components [33,34]. In addition to its roles in replication and membrane remodeling, 6K1 has also been reported to promote infection by interfering with host defense factors. For example, PVY 6K1 can weaken antiviral activity by competitively binding to Nb14-3-3h [35]. It is therefore possible that 6K1 partially interferes with the defense output of GmHrBP1 through direct association at the membrane interface, thereby weakening HrBP1-mediated restriction of SMV.

By contrast, GIP2 was the only factor assigned to the PQC module. Its homolog GmGIP1 restricts *Phytophthora sojae* infection by inactivating the xyloglucanase activity of the effector PsXEG1 [36]. However, HC-Pro, which interacts with GIP2, is not a hydrolase, and the GIP2–HC-Pro interaction was detected in membrane-associated compartments. This suggests that the antiviral effect of GIP2 on SMV is more likely to depend on interference with HC-Pro-associated membrane functions than on enzyme inhibition analogous to that described for GIP1.

GmSTOP1 offers a possible entry point for understanding how SMV may interfere with host transcriptional regulation. As a nuclear-localized C2H2-type zinc finger transcription factor, STOP1 is regulated by SUMOylation, which affects both its stability and transcriptional output [37]. During potyvirus infection, SUMOylation has also been recognized as an important mechanism by which viruses suppress host immunity. For example, TuMV NIb can not only exploit SUMOylation to alter its own localization in favor of replication, but can also suppress PR gene expression by preventing NPR1 SUMOylation and phosphorylation [18,38]. STOP1 showed a clear infection response in both the public RNA-seq dataset and our RT-qPCR validation. Together with the observation that co-expression of 6K2 weakened the antiviral effect of STOP1 and that their interaction occurred in both the nucleus and membrane-associated compartments, these results suggest that 6K2 may compromise STOP1-mediated restriction. Whether this occurs through effects on STOP1-associated transcriptional regulation, post-translational modification, or subcellular distribution remains to be determined.

GmSiPPase may represent a restriction factor linked to host metabolic homeostasis. Downregulation of plastidial soluble inorganic pyrophosphatase has been reported to strongly perturb central chloroplast metabolism, and in potato spindle tuber viroid (PSTVd), silencing of SiPPase also aggravates disease-associated phenotypes [39,40]. Given that the SMV interactome identified here was enriched in chloroplast-associated and basal metabolic processes, the restrictive effect of GmSiPPase on SMV may be associated with the maintenance of chloroplast metabolic homeostasis. The further reduction in SMV accumulation observed upon GmSiPPase and NIa-Pro co-expression was unexpected and may be explained in at least two, non-mutually exclusive ways. First, their association may disturb a virus-favorable metabolic state, thereby enhancing the restrictive effect associated with GmSiPPase. Second, the altered metabolic state caused by this interaction may be sensed by the host and trigger stronger local defense output. A related example has been described for the TRV 16K protein, whose interaction with the Cajal body protein coilin activates SA-mediated defense and limits systemic infection [41]. Thus, under certain conditions, virus–host interactions may enhance, rather than simply suppress, host resistance. However, because NIa-Pro is a viral protease, we cannot exclude the possibility that it affects GmSiPPase stability or processing. Our in silico analysis did not identify an exact match to the reported SMV NIa-Pro cleavage sequence or to general potyvirus NIa-Pro consensus motifs in GmSiPPase, but noncanonical cleavage or protease-dependent destabilization remains possible. Future protein-level assays, such as co-expression followed by Western blotting, co-immunoprecipitation (co-IP) combined with Western blotting, and cleavage-site mutagenesis, will be required to determine whether NIa-Pro affects GmSiPPase stability or processing and to distinguish this possibility from the metabolic-homeostasis-related hypotheses proposed above. Overall, these four representative factors reflect distinct layers of host restriction, including the membrane-associated infection interface, protein homeostasis, host transcriptional regulation, and the chloroplast metabolic environment. They not only correspond to the key host processes highlighted by the interaction network, but also suggest that these processes may contribute functionally to SMV accumulation and systemic infection.

Although this study provides a relatively systematic framework for SMV–soybean interactions, the biological significance of many host factors remains to be resolved given the scale and complexity of the interaction dataset. At present, our understanding of the representative host factors is still based mainly on initial antiviral phenotyping, and the precise molecular mechanisms by which they restrict SMV infection will require deeper analysis integrating protein modification, genetic approaches, metabolic assays, and molecular characterization. Taken together, this study extends the current view of SMV–soybean interactions from scattered analyses of individual viral proteins or host factors to a broader framework in which multiple viral proteins target different host processes, and provides a basis for future exploration and use of antiviral gene resources.

## 4. Materials and Methods

### 4.1. Plant Growth Conditions and Experimental Materials

The susceptible soybean (*Glycine max*) cultivar Williams 82 was used for RT-qPCR analysis of the selected genes. The wild-type line 17 of *N. benthamiana* was used for *Agrobacterium*-mediated transient expression and subcellular localization analyses. Soybean and *N. benthamiana* seeds were surface-sterilized with 10% sodium hypochlorite for 10 min, rinsed three times with sterile distilled water, and then sown in a 1:1 (*v*/*v*) mixture of nutrient soil and vermiculite. All plant materials were grown in an artificial climate chamber under the following conditions: 25 °C, 55–65% relative humidity, and a 16 h light/8 h dark photoperiod. GFP-tagged viral infectious clones (SMV-GFP) was generated in a previous study and had been maintained in our laboratory [42]. The soybean cDNA library used for Y2H screening was constructed by Ouyi Biomedical Technology Co., Ltd. (Shanghai, China).

### 4.2. Sequence Analysis, Gene Cloning, and Vector Construction

Reference sequences of 10 candidate soybean genes were retrieved from the National Center for Biotechnology Information (NCBI) database. Total RNA was extracted from Williams 82 leaves, and first-strand cDNA was used as the template for amplification of the full-length open reading frames (ORFs) of each candidate gene. PCR products were inserted into the corresponding expression vectors by seamless cloning. The bait vector pGBKT7-SMV and its component-derived constructs used for Y2H screening were generated and maintained in our laboratory. Candidate genes were individually cloned into the pGADT7 vector to test their physical interactions with SMV proteins. For *Agrobacterium*-mediated transient expression, candidate gene ORFs were cloned into the SacI/KpnI sites of the binary vector pCambia1300 under the control of the *cauliflower mosaic virus* (CaMV) 35S promoter. For subcellular localization assays, the ORFs were inserted into the pNC-Green-SubC vector. For BiFC assays, the ORFs were cloned into the pNC-BiFC-Ecc vector carrying the cEYFP tag or the pNC-BiFC-Enc vector carrying the nEYFP tag.

### 4.3. Bioinformatics Prediction and Functional Enrichment Analysis

UpSet plots and alluvial diagrams were generated using the ChiPlot online platform https://www.chiplot.online/ (accessed on 21 November 2025) visualize overlap patterns and functional associations within the dataset. The subcellular localization of the 147 candidate proteins was predicted using the WoLF PSORT online tool https://wolfpsort.hgc.jp/ (accessed on 7 November 2025) in batch mode. GO enrichment and KEGG pathway analyses were performed using the OE Cloud platform https://cloud.oebiotech.cn/ (accessed on 14 December 2025). The 2 kb upstream sequences of the translation start codon (ATG) of the 147 candidate genes were extracted using TBtools-II (v2.423) [43], and cis-acting elements in their promoter regions were identified using the PlantCARE database https://bioinformatics.psb.ugent.be/webtools/plantcare/html/ (accessed on 1 March 2026). Protein interaction relationships among the 147 candidate proteins were predicted using the STRING database https://cn.string-db.org (accessed on 20 April 2026) with a confidence score cutoff of ≥0.7. The interaction network was visualized using Cytoscape (v3.10.4), and additional nodes were manually incorporated and functionally categorized to provide a complete view of the distribution of the 147 candidate host proteins.

### 4.4. Acquisition and Expression Analysis of Public Transcriptome Data

Public transcriptome datasets for the susceptible soybean cultivar Williams 82 (SRR5466715–SRR5466722) and the resistant cultivar L29 (SRR10098755–SRR10098764) were retrieved from the NCBI Sequence Read Archive (SRA) database. Data format conversion and sequence quality assessment were performed using SRA Toolkit (v3.0.7) and FastQC (v0.11.9), respectively. High-quality reads were then pseudoaligned and quantified against the Williams 82 reference transcriptome (*Glycine max* Wm82.a4.v1) from Phytozome v14 using Kallisto (v0.46.2) [44]. Expression values for each host gene at different time points after SMV inoculation (mock, 2, 4, 6, and 8 hpi) were extracted and normalized as TPM (transcripts per million) values for comparison between resistant and susceptible cultivars.

### 4.5. RNA Extraction and RT-qPCR Analysis

Total RNA was extracted using TRIzol reagent, and first-strand cDNA was synthesized using the HiScript III RT SuperMix for qPCR kit (Vazyme, Nanjing, China). RT-qPCR was performed on a CFX96 real-time PCR system (Bio-Rad, Hercules, CA, USA) using ChamQ Universal SYBR qPCR Master Mix (Vazyme, Q711).

For soybean expression analysis, leaves of Williams 82 were collected at mock, 2, 4, 6, and 8 h after SMV inoculation. *GmActin* was used as the internal reference, and relative transcript levels were calculated using the 2^−ΔΔCt^ method. Pearson correlation analysis between the RNA-seq data shown in Figure 3C (TPM values) and the RT-qPCR data shown in Figure 3C (relative expression levels) was performed using the ChiPlot online tool. The paired expression heatmaps shown in Figure 3D were generated using TBtools, and the data were row-scaled before visualization.

For SMV accumulation assays in *N. benthamiana*, leaf samples were collected at 5 dpi for the transient co-expression assay shown in Figure 4C, at 25 dpi for the systemic-infection inhibition assay shown in Figure 5D, and at 5 dpi for the viral protein co-expression assay shown in Figure 5F. SMV RNA accumulation was quantified by RT-qPCR using primers targeting the SMV coat protein (CP) coding region, with *NbActin* used as the internal reference. For Figure 5D, SMV RNA accumulation was calculated as the left/right ratio using paired samples from the two halves of the same leaf. All RT-qPCR analyses included three biological replicates, each with three technical replicates.

### 4.6. Pairwise Y2H Validation Assay

Pairwise Y2H assays were performed to validate interactions between selected host candidates and SMV proteins. The pGBKT7 bait plasmids and pGADT7 prey plasmids were co-transformed into the yeast strain Y2HGold using the standard PEG/LiAc transformation method. Positive yeast transformants carrying the recombinant plasmids were first selected on minimal medium lacking leucine and tryptophan (SD/–Leu/–Trp; DDO). The cotransformants were then serially diluted and plated on more stringent selective media, including minimal medium lacking leucine, tryptophan, and histidine (SD/–Leu/–Trp/–His; TDO) and minimal medium additionally lacking adenine (SD/–Leu/–Trp/–His/–Ade; QDO). When indicated, X-α-Gal and aureobasidin A (AbA) were added to the selective media to monitor reporter activation and increase selection stringency. Plates were incubated at 30 °C for 4–5 days, and protein interactions were evaluated based on yeast growth and colony color development.

### 4.7. Y2H Library Screening

Yeast two-hybrid library screening was performed using the mating method. The soybean cDNA AD library had an estimated capacity of 5–6 × 10^5^ cfu. Before mating, the AD prey library was checked by dilution plating on SD/–Leu medium and met the required density of >2 × 10^7^ cells/mL. For each screen, Y2HGold cells carrying a BD-fused SMV bait and Y187 cells carrying the soybean cDNA AD library were mixed and incubated to allow yeast mating. After mating, serial dilutions of the mating mixture were plated on SD/–Trp, SD/–Leu, and SD/–Leu/–Trp (DDO) plates to estimate mating efficiency and the number of screened diploid clones. For each bait, the number of screened diploid clones exceeded the recommended minimum of 1 × 10^6^.

The remaining mating mixture was plated on SD/–Leu/–Trp/–His/X-α-Gal/AbA (TDO/X/A) medium for primary selection. Blue colonies from TDO/X/A plates were transferred with sterile toothpicks to SD/–Leu/–Trp/–His/–Ade/X-α-Gal/AbA (QDO/X/A) medium for at least two rounds of high-stringency re-screening. Colonies that failed to grow reproducibly or failed to activate the reporter on QDO/X/A were excluded. Stable QDO/X/A-positive colonies were further screened by colony PCR, and PCR products were separated by agarose gel electrophoresis. Band sizes were estimated using the Vilber Fusion Solo 6S Edge imaging system (Vilber Lourmat, Collégien, France) and its associated gel-analysis software, and colonies with similar banding patterns were removed as putative duplicates before prey-insert sequencing. After sequence-based filtering and removal of redundant entries, 200 stable positive clones corresponding to 147 nonredundant candidate host proteins were obtained.

### 4.8. Agrobacterium-Mediated Transient Expression and Antiviral Function Assays

Recombinant plasmids were introduced into *Agrobacterium* tumefaciens strain GV3101 by the freeze–thaw method. Cultures were grown at 28 °C with shaking to the logarithmic growth phase, collected by centrifugation, and resuspended in infiltration buffer (10 mM MES-KOH, pH 5.6; 10 mM MgCl_2_; 100 μM acetosyringone). After incubation at room temperature in the dark for 3 h, the suspensions were used for infiltration.

For initial evaluation of antiviral activity, *Agrobacterium* carrying the target gene was mixed in equal volumes with *Agrobacterium* carrying SMV-GFP or TMV-GFP infectious clones (final OD_600_ = 1) and infiltrated into separate local regions of *N. benthamiana* leaves using a 1 mL needleless syringe. At 5 dpi, viral fluorescence in infiltrated regions was examined under a handheld UV lamp [45,46]. To assess the effects of candidate genes on systemic spread of SMV, *Agrobacterium* carrying the candidate gene was co-infiltrated with SMV-GFP into the basal leaves of *N. benthamiana*. The systemic spread of SMV to upper newly emerged tissues was monitored at 5, 10, 15, and 20 dpi.

For assays performed after systemic infection had been established, SMV-GFP was first inoculated into the basal leaves of *N. benthamiana*. At 7 dpi, when the virus had spread to upper young tissues, *Agrobacterium* carrying the antiviral gene was infiltrated into the left region of a subapical leaf, whereas the right region served as an internal control. SMV accumulation in the two half-leaves was compared at 25 dpi. For fluorescence-based quantification, GFP-positive areas were measured using ImageJ from the green channel after applying the same threshold settings to all images. The GFP-positive area was normalized to the total area of the corresponding half-leaf region, and the left/right ratio was calculated for each biological replicate. To determine how viral proteins affected host restriction factors, *Agrobacterium* carrying the host gene, the corresponding interacting SMV protein, or both constructs together was infiltrated into separate regions of the same *N. benthamiana* leaf. At 1 dpi, SMV-GFP was uniformly inoculated into these regions, and local viral accumulation was assessed at 5 dpi.

### 4.9. Subcellular Localization and BiFC Assays

The *Agrobacterium*-mediated transient expression system described above was used for subcellular localization and BiFC analyses [47]. For BiFC assays, *Agrobacterium* cultures carrying the nEYFP and cEYFP fusion constructs were mixed in equal volumes (final OD_600_ = 1) and co-infiltrated into *N. benthamiana* leaves. For subcellular localization assays, *Agrobacterium* carrying the eGFP fusion construct was infiltrated individually into *N. benthamiana* leaves. At 48–72 hpi, leaf samples were collected and examined with a confocal laser scanning microscope. EYFP fluorescence was detected with 513 nm excitation and 520–550 nm emission. eGFP fluorescence was detected with 488 nm excitation and 500–530 nm emission. To label cellular structures, LTI6b-mCherry was used as a plasma membrane marker, whereas Histone3-mCherry transgenic *N. benthamiana* plants or DAPI (1 μg/mL; excitation 358 nm, emission 461 nm) was used to label the nucleus. mCherry signals were detected with 587 nm excitation and 600–630 nm emission. Chloroplasts were visualized by chlorophyll autofluorescence in the far-red channel, which was detected at 637–720 nm.

### 4.10. Statistical Analysis

All experiments were performed with at least three independent biological replicates. Statistical analyses were carried out using GraphPad Prism (v9.0). Comparisons among multiple groups were conducted using one-way ANOVA followed by Dunnett’s multiple-comparison test, with the EV group as the control. Data are presented as mean ± SD. Statistical significance is indicated as follows:* *p* < 0.05, ** *p* < 0.01, *** *p* < 0.001.

## Data Availability

The publicly available RNA-seq datasets reanalyzed in this study are available from the NCBI Sequence Read Archive (SRA) under accession numbers SRR5466715–SRR5466722 (Williams 82) and SRR10098755–SRR10098764 (L29). All other data generated or analyzed during this study are included in this article and its Supplementary Information.

## Supplementary Materials

The following supporting information can be downloaded at: https://www.mdpi.com/article/10.3390/plants15111650/s1, Table S1: Candidate host proteins identified with each of the 10 SMV proteins as bait; Table S2: 147 nonredundant candidate host proteins identified from screening with the 10 SMV proteins; Table S3: Functional backgrounds and literature-supported rationale for the 10 candidate host proteins selected for validation; Table S4: Exact adjusted *p*-values for RT-qPCR quantification shown in Figure 4C and Figure 5D,F; Table S5: Exact adjusted *p*-values for fluorescence-based quantification shown in Figure S2; Table S6: Cloning and RT-qPCR primers used in this study. Figure S1: Schematic diagrams of the SMV-GFP infectious clone and TMV-GFP (pJL24) vector used in the fluorescence-based assays; Figure S2: Fluorescence-based quantification of SMV-GFP accumulation in transient-expression assays; Figure S3: Reciprocal BiFC validation of the interaction between GmSTOP1 and SMV 6K2. Refs. [33,36,37,39,40,48,49,50,51,52,53,54] are cited in the Supplementary Materials.
