# Supplementary material for: Systematic Screening of Host Interactors for Soybean mosaic virus Proteins Identifies Four Soybean (Glycine max) Antiviral Factors
Source: Plants (Basel). 2026 May 27;15(11):1650. doi: 10.3390/plants15111650 (PMC13259008; doi:10.3390/plants15111650)
Supplement: Supplementary file 1 [file plants-15-01650-s001.zip › supplementary materials/Supplementary Figure S1-3.pdf]

Supplementary Figure S1

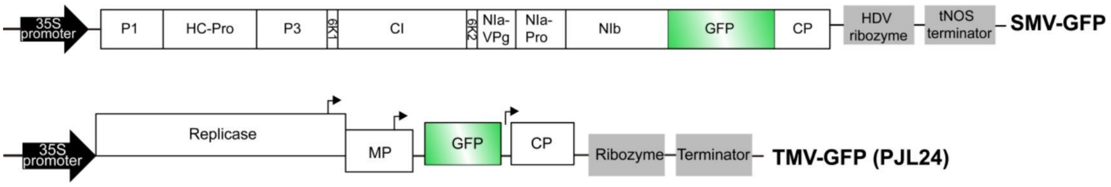

Supplementary Figure S1. Schematic diagrams of the SMV-GFP infectious clone and TMV-GFP (pJL24) vector used in the fluorescence-based assays. In the SMV-GFP infectious clone, the SMV-GFP cDNA is driven by the CaMV 35S promoter and followed by the HDV ribozyme and tNOS terminator within the T-DNA region. GFP was inserted between NIb and CP in the SMV genome. In TMV-GFP (pJL24), GFP is positioned between MP and CP and is expressed from a TMV subgenomic promoter. Bent arrows indicate subgenomic promoters.

Supplementary Figure S2

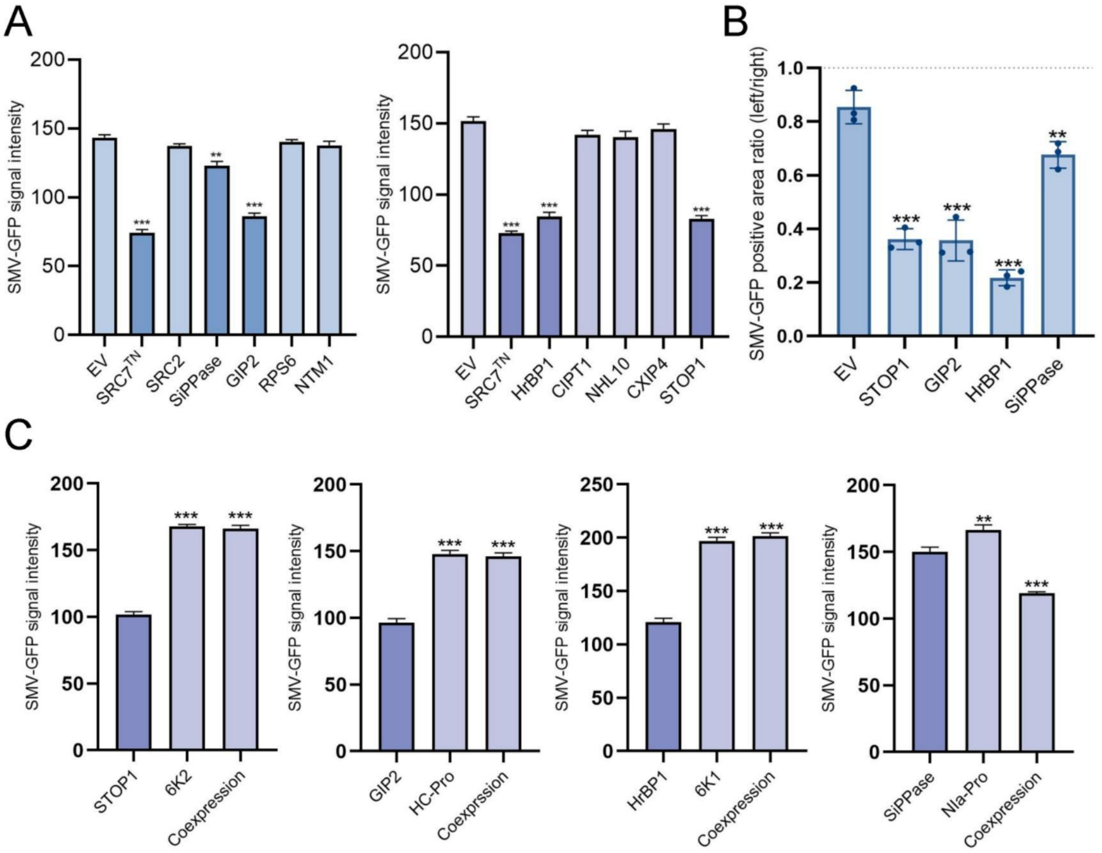

Supplementary Figure S1. Fluorescence-based quantification of SMV-GFP accumulation in transient-expression assays. (A) Quantification of SMV-GFP fluorescence in leaves co-expressing SMV-GFP and each of the 10 candidate genes. Fluorescence intensity was measured from the inf ected leaf areas shown in Fig. 4B using ImageJ. **(B)** Fluorescence-based quantification of SMV-GF P accumulation in the local inhibition assay after systemic infection had been established. GFP-p

ositive areas were measured from the green channelusing ImageJ , normalized to the total area o f the corresponding half-leaf region, and expressed as the left/right ratio. **(C)** Quantification of S MV-GFP fluorescence in the co-expression assay examining the effects of the corresponding SM V proteins on the antiviral activities of GmSTOP1, GmHrBP1, GmSiPPase, and GmGIP2. Fluoresc ence was measured from the infiltrated regions shown in Fig. 5E using ImageJ. Data represent t he mean ± SD of three independent biological replicates. Asterisks indicate significant differences compared with the corresponding control groups (***p* < 0.01, ****p* < 0.001; one-way ANOVA foll owed by Dunnett's multiple-comparison test).

Supplementary Figure S3

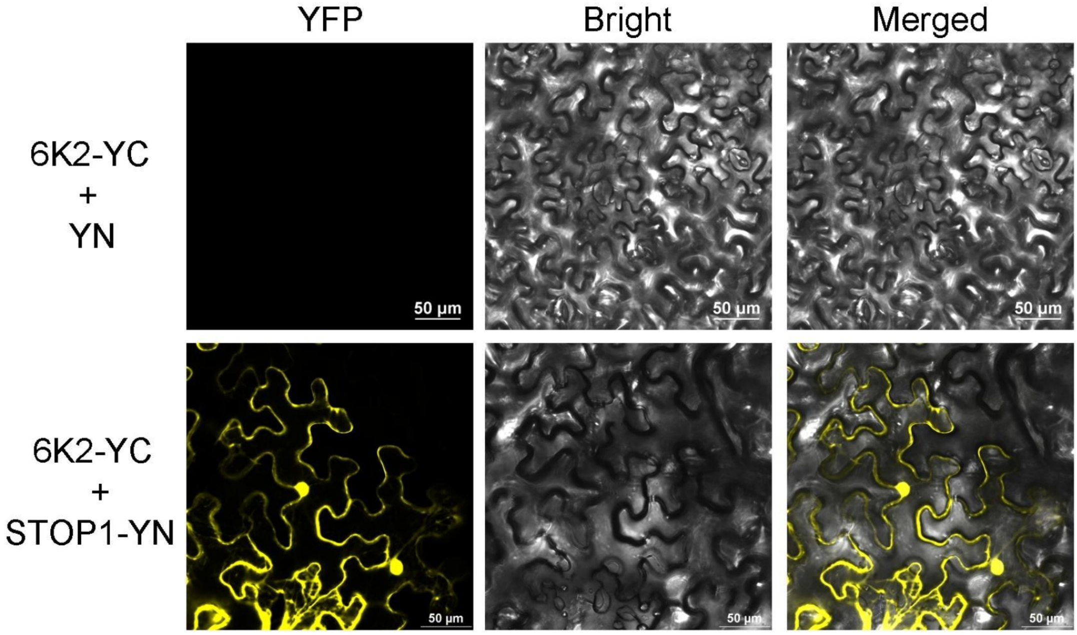

Supplementary Figure S3. Reciprocal BiFC validation of the interaction between GmSTOP 1 and SMV 6K2. GmSTOP1 and 6K2 were fused to the N- or C- terminal fragments of YFP in reciprocal orientations and transiently co-expressed in *N. benthamiana* leaves. The reciprocal comb ination GmSTOP1-YN + 6K2-YC produced detectable YFP fluorescence mainly around the nuclea r envelope/perinuclear region, whereas the corresponding empty-vector controls showed no detec table fluorescence. At 48 hpi, YFP fluorescence was examined by confocal microscopy. EYFP sign als were detected with excitation at 514 nm and emission at 520–550 nm.
