# Supplementary material for: Systematic Screening of Host Interactors for Soybean mosaic virus Proteins Identifies Four Soybean (Glycine max) Antiviral Factors
Source: Plants (Basel). 2026 May 27;15(11):1650. doi: 10.3390/plants15111650 (PMC13259008; doi:10.3390/plants15111650)
Supplement: Supplementary file 1 [file plants-15-01650-s001.zip › supplementary materials/Supplementary Table S1.pdf]

Supplementary Table S1. Candidate host proteins identified with each of the 10 SMV proteins as bait. Candidate factors were grouped according to the viral bait protein used in yeast two-h ybrid screening. Soybean gene IDs and their corresponding annotation descriptions are listed as obtained from the screening results. Entries with identical annotation descriptions but different g ene IDs were retained as independent candidate factors.

Candidate interacting proteins of 6K1

Gene ID	Description
Glyma.06G076000	COP9 signalosome complex subunit 5a
Glyma.09G105600	Carbon catabolite repressor protein 4 homolog 1
Glyma.14G102700	Chorismate mutase 2
Glyma.20G207100	Harpin binding protein 1(HRBP1)
Glyma.08G164100	Inosine-5'-monophosphate dehydrogenase
Glyma.07G216900	Proline-rich antigen homolog
Glyma.08G206000	Translation machinery associated TMA7 superfamily protein
Glyma.17G073300	Signal recognition particle receptor subunit alpha
Glyma.11G213600	Cytochrome c oxidase assembly protein COX11

Candidate interacting proteins of CP

Gene ID	Description
Glyma.06G059600	Zinc finger protein CONSTANS-LIKE 4
Glyma.17G099300	Activating signal cointegrator 1
Glyma.16G008900	Subtilisin-like protease SBT1.4
Glyma.07G117000	ATP synthase subunit delta
Glyma.19G011900	RNA ligase/cyclic nucleotide phosphodiesterase family protein
Glyma.18G192800	Phosphatidylinositol:ceramide inositolphosphotransferase 1
Glyma.04G020300	Plastocyanin
Glyma.06G020400	Putative plastocyanin

Glyma.08G209100	ADP，ATP carrier protein 1

Candidate interacting proteins of NIa-VPg

Gene ID	Description
Glyma.13G224000	Heat shock 70 kDa protein-like
Glyma.04G020300	Plastocyanin
Glyma.19G106800	Glyceraldehyde-3-phosphate dehydrogenase A subunit
Glyma.11G045100	Transcription factor ABIG1-like protein
Glyma.16G044900	Glyceraldehyde-3-phosphate dehydrogenase A, chloroplastic
Glyma.13G356700	Pentatricopeptide repeat-containing protein At3g02650, mitochondrial
Glyma.17G037900	Leghemoglobin reductase-like
Glyma.03G105300	ATP synthase subunit delta', mitochondrial
Glyma.10G079900	NDR1/HIN1-like protein 10
Glyma.01G139600	3-phosphoshikimate 1-carboxyvinyltransferase 2
Glyma.10G170200	Protein PELOTA 1
Glyma.02G075100	Sucrose transport protein SUC8
Glyma.18G125500	Mitochondrial outer membrane protein porin of 36 kDa-like
Glyma.20G103700	E3 ubiquitin-protein ligase RING1
Glyma.14G048800	Vacuolar sorting protein
Glyma.20G219800	Protein pelota-like
Glyma.03G028800	Methionine aminopeptidase 1A
Glyma.10G284600	CAX-interacting protein 4
Glyma.17G039100	S-adenosylmethionine synthase-like
Glyma.14G206100	Root phototropism protein 2
Glyma.17G197700	DNA ligase 1

Glyma.03G010800	UPF0187 protein At3g61320
Glyma.19G227700	Chloroplast stem-loop binding protein of 41 kDa b
Glyma.07G117000	ATP synthase subunit delta', mitochondrial
Glyma.06G007300	Probable ribosome-binding factor A
Glyma.15G004800	Uncharacterized LOC100811711
Glyma.06G015900	Glyceraldehyde-3-phosphate dehydrogenase B subunit
Glyma.07G118700	Methyl-CpG-binding domain-containing protein
Glyma.04G075000	COP9 signalosome complex subunit 5a
Glyma.06G059600	Zinc finger protein CONSTANS-LIKE 4
Glyma.15G064300	Multiple organellar RNA editing factor 9
Glyma.07G142000	Phosphatidylinositol:ceramide inositolphosphotransferase 1
Glyma.15G268100	E3 ubiquitin-protein ligase RNF181
Glyma.16G008900	Subtilisin-like protease SBT1.4
Glyma.13G163700	UPF0051 protein ABCI8
Glyma.13G208200	Probable aspartic proteinase GIP2
Glyma.19G124800	E3 ubiquitin-protein ligase At4g11680
Glyma.06G076000	COP9 signalosome complex subunit 5a
Glyma.06G020400	Putative plastocyanin (LOC100499708), mRNA
Glyma.03G044500	Dirigent protein 22
Glyma.12G225500	Proline-rich extensin-like protein EPR1
Glyma.02G178800	Protein EMSY-LIKE 1
Glyma.18G192800	Phosphatidylinositol:ceramide inositolphosphotransferase 1
Glyma.02G024200	Uncharacterized LOC100797561
Glyma.07G056900	Abscisic acid receptor PYL9-like
Glyma.05G177100	Ahaperone protein dnaJ A6

Candidate interacting proteins of NIa-Pro

Gene ID	Description
Glyma.03G172700	Soluble inorganic pyrophosphatase
Glyma.06G020400	Putative plastocyanin
Glyma.11G111600	Uncharacterized LOC100820348
Glyma.12G169400	Ferredoxin-A
Glyma.01G196600	Homeobox-leucine zipper protein HAT22
Glyma.09G210900	Phosphoribulokinase
Glyma.08G134300	Haloacid dehalogenase-like hydrolase domain-containing protein
Glyma.04G008300	Fructose-bisphosphate aldolase 1, chloroplastic
Glyma.11G061300	Oxygen-evolving enhancer protein 1
Glyma.20G103400	Transcription factor TCP7
Glyma.19G106800	Glyceraldehyde-3-phosphate dehydrogenase A subunit
Glyma.09G023700	Ubiquitin-40S ribosomal protein S27a
Glyma.07G020300	RNA-binding KH domain-containing protein RCF3
Glyma.13G210800	Glutamine synthetase precursor (GS-2)
Glyma.03G190600	Splicing factor U2af small subunit B
Glyma.06G076000	COP9 signalosome complex subunit 5a
Glyma.17G052100	WD repeat-containing protein VIP3
Glyma.04G075000	COP9 signalosome complex subunit 5a
Glyma.15G104900	Probable aspartic proteinase GIP2
Glyma.05G018300	Isoaspartyl peptidase/L-asparaginase-like
Glyma.08G239900	Patellin-3
Glyma.05G156300	Protochlorophyllide-dependent translocon component 52

Candidate interacting proteins of NIb

Gene ID	Description
Glyma.04G020300	Plastocyanin
Glyma.19G011900	RNA ligase/cyclic nucleotide phosphodiesterase family protein
Glyma.17G099300	Activating signal cointegrator 1
Glyma.05G177100	Chaperone protein dnaJ A6
Glyma.04G075000	COP9 signalosome complex subunit 5a
Glyma.14G031800	Oxygen-evolving enhancer protein
Glyma.04G230000	Uncharacterized LOC100527213
Glyma.06G020400	Putative plastocyanin
Glyma.06G059600	Zinc finger protein CONSTANS-LIKE 4
Glyma.08G024100	Uncharacterized LOC100788535
Glyma.11G233700	Biotin carboxyl carrier protein of acetyl-CoA carboxylase-like
Glyma.10G265400	Probable ribose-5-phosphate isomerase 2
Glyma.08G265400	Uncharacterized LOC100820080
Glyma.17G130100	ATP synthase delta chain
Glyma.13G338600	Curved DNA-binding protein
Glyma.16G044900	Glyceraldehyde-3-phosphate dehydrogenase A
Glyma.08G090300	TRNA-dihydrouridine(20/20a) synthase
Glyma.05G010800	Uncharacterized LOC100306194
Glyma.05G237200	Protein SAR DEFICIENT 1
Glyma.16G008900	Subtilisin-like protease SBT1.4
Glyma.16G010000	Protein TIFY 10A-like
Glyma.15G268100	E3 ubiquitin-protein ligase RNF181
Glyma.01G196600	Homeobox-leucine zipper protein HAT22
Glyma.17G058700	flocculation protein FLO11-like

Glyma.03G105300	ATP synthase subunit delta'
Glyma.04G015900	Glyceraldehyde-3-phosphate dehydrogenase B

Candidate interacting proteins of P1

Gene ID	Description
Glyma.06G020400	Putative plastocyanin
Glyma.16G044900	Glyceraldehyde-3-phosphate dehydrogenase A
Glyma.16G008900	Subtilisin-like protease SBT1_4
Glyma.04G020300	Plastocyanin
Glyma.19G106800	Glyceraldehyde-3-phosphate dehydrogenase A subunit
Glyma.07G142000	Phosphatidylinositol: ceramide inositol phosphotransferase 1
Glyma.04G230000	Uncharacterized LOC100527213
Glyma.04G015900	Glyceraldehyde-3-phosphate dehydrogenase B
Glyma.04G075000	COP9 signalosome complex subunit 5a
Glyma.18G192800	Phosphatidylinositol ceramide inositol phosphotransferase 1
Glyma.08G321100	Aspartyl protease family protein

Candidate interacting proteins of P3

Gene ID	Description
Glyma.19G123800	Peroxisomal voltage-dependent anion-selective channel protein
Glyma.20G169500	Light-harvesting complex-like protein OHP2
Glyma.08G011100	Heavy metal-associated isoprenylated plant protein 7
Glyma.17G062500	VIN3-like protein 2
Glyma.10G005200	Cyanate hydratase (CYN)
Glyma.18G028400	Chlorophyll a-b binding protein CP29_3
Glyma.02G079400	Light-harvesting complex-like protein OHP2

Glyma.05G183500	Stress enhanced protein 2
Glyma.02G161100	BZIP transcription factor (BZIP68)
Glyma.20G170700	Ran-binding protein 1 homolog b
Glyma.10G197600	NAC domain-containing protein 78
Glyma.05G058700	Vesicle-associated protein
Glyma.11G113800	Uncharacterized LOC100784194
Glyma.19G131200	Sec14p-like lipid-binding domain-containing protein
Glyma.06G059600	CONSTANS-like zinc finger protein (COL3)
Glyma.11G135900	Plastid transcriptionally active 16

Candidate interacting proteins of CI

Gene ID	Description
Glyma.09G240600	Peroxisomal voltage dependent anion selective channel protein
Glyma.06G003800	MYB transcription factor (MYB48)
Glyma.17G106000	Ubiquitin-like domain-containing protein CIP73
Glyma.20G219800	Protein pelota-like
Glyma.10G005200	Cyanate hydratase (CYN)
Glyma.01G010200	Phosphoribulokinase
Glyma.04G193500	Glyceraldehyde 3-phosphate dehydrogenase (GAPDH)
Glyma.16G044900	Glyceraldehyde-3-phosphate dehydrogenase A
Glyma.06G150300	Uncharacterized LOC100306036
Glyma.11G044600	Cysteine desulfurase
Glyma.17G188300	Uncharacterized LOC100775929
Glyma.17G039100	S-adenosylmethionine synthase-like
Glyma.19G106800	Glyceraldehyde-3-phosphate dehydrogenase A subunit
Glyma.12G037400	Putative fructose-bisphosphate aldolase 2

Glyma.04G008300	Fructose-bisphosphate aldolase 1
Glyma.02G132100	Nitrate reductase (NIR)
Glyma.08G274400	Serine hydroxymethyltransferase3
Glyma.08G138200	Inositol-3-phosphate synthase (MIPS4)
Glyma.18G192800	Phosphatidylinositol ceramide inositol phosphotransferase 1
Glyma.04G075000	COP9 signalosome complex subunit 5a
Glyma.07G013300	Peptidase M20 dimerisation domain-containing protein
Glyma.09G154700	Chlorophyll a-b binding protein CP26

Candidate interacting proteins of 6K2

Gene ID	Description
Glyma.06G076000	COP9 signalosome complex subunit 5a
Glyma.04G075000	COP9 signalosome complex subunit 5a
Glyma.05G162700	SEI2A mRNA for seipin 2A
Glyma.20G123200	Uncharacterized LOC100800265
Glyma.18G030000	Protein SRC2 homolog
Glyma.04G216700	Mediator of RNA polymerase II transcription subunit
Glyma.16G076800	Glycine and proline rich protein3(GPRP3)
Glyma.08G356700	Tetratricopeptide repeat protein1
Glyma.09G117900	Auxin efflux carrier component3d(PIN3D)
Glyma.02G019000	Binding partner of ACD11
Glyma.04G196200	Nuclear factor Y transcription factor family protein (NF-YC03)
Glyma.05G055100	Non-specific lipid transfer protein GPI-anchored7
Glyma.20G014300	Auxin efflux carrier component3b(PIN3B)
Glyma.01G195500	ACT domain-containing protein ACR1
Glyma.14G189300	NTM1-like9

Glyma.18G151800	Ribosomal proteinS6(RPS6)
Glyma.04G095200	Uncharacterized LOC100798145
Glyma.20G192300	NAC domain-containing protein53
Glyma.10G215200	C2-H2 zinc finger protein (STOP1)
Glyma.16G109900	Uncharacterized LOC100305832
Glyma.10G005200	Cyanate hydratase (CYN)
Glyma.17G141100	Zinc finger protein CONSTANS-LIKE2(COL2B)
Glyma.04G036000	Thylakoidal processing peptidase2
Glyma.11G135900	Plastid transcriptionally active16
Glyma.06G262900	Uncharacterized LOC100782462
Glyma.01G190900	Lysine-rich arabinogalactan protein18
Glyma.06G097000	Uncharacterized LOC100781025
Glyma.17G009200	Uncharacterized LOC100777525
Glyma.03G178800	Cell number regulator8
Glyma.05G058700	Vesicle-associated protein
Glyma.02G222300	NAC domain protein (NAC22)

Candidate interacting proteins of HC-Pro

Gene ID	Description
Glyma.13G208200	Probable aspartic proteinase GIP2
Glyma.09G154700	Chlorophyll a-b binding protein CP26
Glyma.15G006900	Chaperone protein DnaJ
Glyma.12G037400	Fructose-bisphosphate aldolase2
Glyma.04G075000	COP9 signalosome complex subunit5a
Glyma.01G196600	Homeobox-leucine zipper protein HAT22
Glyma.12G169400	Ferredoxin-A

Glyma.13G366300	Chaperone protein dnaJA6
