# Supplementary material for: Systematic Screening of Host Interactors for Soybean mosaic virus Proteins Identifies Four Soybean (Glycine max) Antiviral Factors
Source: Plants (Basel). 2026 May 27;15(11):1650. doi: 10.3390/plants15111650 (PMC13259008; doi:10.3390/plants15111650)
Supplement: Supplementary file 1 [file plants-15-01650-s001.zip › supplementary materials/Supplementary Table S3-5.pdf]

Supplementary Table 3. Functional backgrounds and literature-supported rationale for the 10 candidate host proteins selected for validation. The listed references support the biological relevance of each candidate for initial validation. The 10 candidates were sel ected based on their primary functional backgrounds and literature-supported relevance to host responses during infection. They we re not intended to statistically represent all 147 nonredundant candidates, but to provide a biologically informed validation set span ning several functional backgrounds.

Candidate protein	Functional basis for prioritization	Functional module	Literature-supported relevance
**HrBP1**	Harpin-binding protein linked to antiviral and SA-associated defense	Defense and stress adaptation	HrBP1 is a dufulin-responsive target that activates SA-related antiviral responses in tobacco.[1]
**CIPT1**	IPCS-like enzyme involved in sphingolipid biosynthesis and defense-associated PCD	Biosynthesis and specialized metabolism	Arabidopsis IPCS/ERH1 has been linked to defense-associate d programmed cell death and RPW8-related HR-like respons es.[2]
**CXIP4**	CAX-interacting protein linked to Ca^{2+} tran sport and stress-related Ca^{2+} regulation	Development, transport and signaling	VvCXIP4 regulates CAX-mediated Ca^{2+} homeostasis, and TaH RC-related studies suggest a possible link between CAXIP4 a nd calcium-mediated immune regulation[3,4]
**NHL10**	NDR1/HIN1-like protein associated with H R and virus/pathogen responses	Defense and stress adaptation	Arabidopsis NHL10 is induced during the hypersensitive res ponse to Cucumber mosaic virus infection.[5]
**SiPPase**	Plastidial soluble pyrophosphatase linked t o chloroplast metabolic homeostasis	Photosynthesis and energy metabolism	Plastidial SiPPase is required for chloroplast central metaboli sm and has been linked to viroid-associated symptoms.[6,7]
**GIP2**	Protease-related glucanase inhibitor protein linked to pathogen–host enzymatic interfa ces	Protein quality control and degradation	Soybean GmGIP1/XEGIP-like proteins bind pathogen effectors and interfere with effector-associated virulence.[8]
**SRC2**	C2-domain Ca^{2+}/lipid-binding protein linke d to membrane-associated stress defense	Development, transport and signaling	A pepper SRC2 homolog is implicated in resistance to host and non-host pathogens.[9]
**NTM1**	Membrane-tethered NAC transcription fact or linked to effector-targeted immune regu lation	Nucleic acids and gene expression	The *Pseudomonas syringae* effector HopD1 targets Arabidopsis NTL9 and suppresses ETI-associated gene induction.[10]
**RPS6**	Ribosomal protein linked to translation co ntrol during plant RNA virus infection	Nucleic acids and gene expression	Silencing of RPS6 in *N. benthamiana* alters infection by plant RNA viruses, supporting its role as a translation-related host factor.[11]
**STOP1**	C2H2 zinc-finger transcription factor linked to stress-responsive gene regulation	Nucleic acids and gene expression	STOP1/STOP1-like proteins regulate multiple stress responses, and STOP1 stability is controlled by SUMOylation.[12]

1. Chen, Z.; Zeng, M.; Song, B.; Hou, C.; Hu, D.; Li, X.; Wang, Z.; Fan, H.; Bi, L.; Liu, J.; et al. Dufulin activates HrBP1 to produce antiviral responses in tobacco. *PLoS One* **2012**, *7*, e37944, doi:10.1371/journal.pone.0037944.
2. Wang, W.; Yang, X.; Tangchaiburana, S.; Ndeh, R.; Markham, J.E.; Tsegaye, Y.; Dunn, T.M.; Wang, G.L.; Bellizzi, M.; Parsons, J.F.; et al. An inositolphosphorylceramide synthase is involved in regulation of plant programmed cell death associated with defense in Arabidopsis. *Plant Cell* **2008**, *20*, 3163–3179, doi:10.1105/tpc.108.060053.
3. Martins, V.; Gerós, H. The grapevine CAX-interacting protein VvCXIP4 is exported from the nucleus to activate the tonoplast Ca(2+)/H(+) exchanger VvCAX3 . *Planta* **2020**, *252*, 35, doi:10.1007/s00425-020-03442-x.
4. Chen, H.; Su, Z.; Tian, B.; Hao, G.; Trick, H.N.; Bai, G. TaHRC suppresses the calcium-mediated immune response and triggers wheat Fusarium head blight susceptibility. *Plant Physiol* **2022**, *190*, 1566–1569, doi:10.1093/plphys/kiac352.

5. Zheng, M.S.; Takahashi, H.; Miyazaki, A.; Hamamoto, H.; Shah, J.; Yamaguchi, I.; Kusano, T. Up-regulation of Arabidopsis thaliana NHL10 in the hypersensitive response to Cucumber mosaic virus infection and in senescing leaves is controlled by signalling pathways that differ in salicylate involvement. *Planta* **2004**, *218*, 740–750, doi:10.1007/s00425-003-1169-2.
6. George, G.M.; van der Merwe, M.J.; Nunes-Nesi, A.; Bauer, R.; Fernie, A.R.; Kossmann, J.; Lloyd, J.R. Virus-induced gene silencing of plastidial soluble inorganic pyrophosphatase impairs essential leaf anabolic pathways and reduces drought stress tolerance in Nicotiana benthamiana. *Plant Physiol* **2010**, *154*, 55–66, doi:10.1104/pp.110.157776.
7. Eamens, A.L.; Smith, N.A.; Dennis, E.S.; Wassenegger, M.; Wang, M.B. In Nicotiana species, an artificial microRNA corresponding to the virulence modulating region of Potato spindle tuber viroid directs RNA silencing of a soluble inorganic pyrophosphatase gene and the development of abnormal phenotypes. *Virology* **2014**, *450-451*, 266–277, doi:10.1016/j.virol.2013.12.019.
8. Ma, Z.; Zhu, L.; Song, T.; Wang, Y.; Zhang, Q.; Xia, Y.; Qiu, M.; Lin, Y.; Li, H.; Kong, L.; et al. A paralogous decoy protects *Phytophthora sojae* apoplastic effector PsXEG1 from a host inhibitor. *Science* **2017**, *355*, 710–714, doi:10.1126/science.aai7919.
9. Kim, Y.C.; Kim, S.Y.; Choi, D.; Ryu, C.M.; Park, J.M. Molecular characterization of a pepper C2 domain-containing SRC2 protein implicated in resistance against host and non-host pathogens and abiotic stresses. *Planta* **2008**, *227*, 1169–1179, doi:10.1007/s00425-007-0680-2.
10. Block, A.; Toruño, T.Y.; Elowsky, C.G.; Zhang, C.; Steinbrenner, J.; Beynon, J.; Alfano, J.R. The Pseudomonas syringae type III effector HopD1 suppresses effector-triggered immunity, localizes to the endoplasmic reticulum, and targets the Arabidopsis transcription factor NTL9. *New Phytol* **2014**, *201*, 1358–1370, doi:10.1111/nph.12626.
11. Yang, C.; Zhang, C.; Dittman, J.D.; Whitham, S.A. Differential requirement of ribosomal protein S6 by plant RNA viruses with different translation initiation strategies. *Virology* **2009**, *390*, 163–173, doi:10.1016/j.virol.2009.05.018.
12. Fang, Q.; Zhang, J.; Zhang, Y.; Fan, N.; van den Burg, H.A.; Huang, C.F. Regulation of Aluminum Resistance in Arabidopsis Involves the SUMOylation of the Zinc Finger Transcription Factor STOP1. *Plant Cell* **2020**, *32*, 3921–3938, doi:10.1105/tpc.20.00687.

Supplementary Table S4. Exact adjusted *p*-values for RT-qPCR quantification shown in Figures 4C, 5D, and 5F. Adjusted *p*-values were calculated using one-way ANOVA followed by Dunnett's multiple-comparison test. For Figures 4C and 5D, comparisons were made against the EV control. For Figure 5F, comparisons were made against the corresponding host factor-alone group. The " Significance in figure " column shows the significance label displayed in the corresponding figure panel: * indicates $p < 0.05$, ** indicates $p < 0.01$, and *** indicates $p < 0.001$; ns, not significant.

Fig. panel	Comparison	Readout	Adjusted *p*-value	Significance in figure
Fig. 4C (left)	EV vs. SRC7^{TN}	Relative SMV RNA accumulation	<0.0001	***
Fig. 4C (left)	EV vs. SRC2	Relative SMV RNA accumulation	0.1995	ns
Fig. 4C (left)	EV vs. SiPPase	Relative SMV RNA accumulation	0.0011	**
Fig. 4C (left)	EV vs. GIP2	Relative SMV RNA accumulation	<0.0001	***
Fig. 4C (left)	EV vs. RPS6	Relative SMV RNA accumulation	0.9918	ns
Fig. 4C (left)	EV vs. NTM1	Relative SMV RNA accumulation	0.6115	ns
Fig. 4C (right)	EV vs. SRC7^{TN}	Relative SMV RNA accumulation	<0.0001	***
Fig. 4C (right)	EV vs. HrBP1	Relative SMV RNA accumulation	<0.0001	***
Fig. 4C (right)	EV vs. CIPT1	Relative SMV RNA accumulation	0.3433	ns
Fig. 4C (right)	EV vs. NHL10	Relative SMV RNA accumulation	0.0538	ns
Fig. 4C (right)	EV vs. CXIP4	Relative SMV RNA accumulation	0.9654	ns
Fig. 4C (right)	EV vs. STOP1	Relative SMV RNA accumulation	<0.0001	***
Fig. 5D	EV vs. STOP1	SMV RNA ratio (left/right)	<0.0001	***
Fig. 5D	EV vs. GIP2	SMV RNA ratio (left/right)	<0.0001	***
Fig. 5D	EV vs. HrBP1	SMV RNA ratio (left/right)	<0.0001	***
Fig. 5D	EV vs. SiPPase	SMV RNA ratio (left/right)	<0.0001	***
Fig. 5F	STOP1 vs. 6K2	Relative SMV RNA accumulation	<0.0001	***
Fig. 5F	STOP1 vs. Coexpression	Relative SMV RNA accumulation	<0.0001	***
Fig. 5F	GIP2 vs. HC-Pro	Relative SMV RNA accumulation	<0.0001	***
Fig. 5F	GIP2 vs. Coexprssion	Relative SMV RNA accumulation	<0.0001	***
Fig. 5F	HrBP1 vs. 6K1	Relative SMV RNA accumulation	<0.0001	***
Fig. 5F	HrBP1 vs. Coexpression	Relative SMV RNA accumulation	<0.0001	***
Fig. 5F	SiPPase vs. NIa-Pro	Relative SMV RNA accumulation	<0.0194	*
Fig. 5F	SiPPase vs. Coexpression	Relative SMV RNA accumulation	<0.0001	***

Supplementary Table s. Exact adjusted *p*-values for fluorescence-based quantification shown in Supplementary Figure S2. Adjusted *p*-values were calculated using one-way ANOVA followed by Dunnett's multiple-comparison test. For Figures 4C and 5D-related fluorescence quantification, comparisons were made against the EV control. For Figure 5F-related fluorescence quantification, comparisons were made against the corresponding host factor-alone group. The " Significance in figure " column shows the significance label displayed in the corresponding figure panel: ** indicates $p < 0.01$, and *** indicates $p < 0.001$; ns, not significant.

Fig. panel	Comparison	Readout	Adjusted *p*-value	Significance in figure
Fig. 4C (left)	EV vs. SRC7^{TN}	SMV-GFP signal intensity	<0.0001	***
Fig. 4C (left)	EV vs. SRC2	SMV-GFP signal intensity	0.4889	ns
Fig. 4C (left)	EV vs. SiPPase	SMV-GFP signal intensity	0.0018	**
Fig. 4C (left)	EV vs. GIP2	SMV-GFP signal intensity	<0.0001	***
Fig. 4C (left)	EV vs. RPS6	SMV-GFP signal intensity	0.8681	ns
Fig. 4C (left)	EV vs. NTM1	SMV-GFP signal intensity	0.9998	ns
Fig. 4C (right)	EV vs. SRC7^{TN}	SMV-GFP signal intensity	<0.0001	***
Fig. 4C (right)	EV vs. HrBP1	SMV-GFP signal intensity	<0.0001	***
Fig. 4C (right)	EV vs. CIPT1	SMV-GFP signal intensity	0.3185	ns
Fig. 4C (right)	EV vs. NHL10	SMV-GFP signal intensity	0.1086	ns
Fig. 4C (right)	EV vs. CXIP4	SMV-GFP signal intensity	0.7975	ns
Fig. 4C (right)	EV vs. STOP1	SMV-GFP signal intensity	<0.0001	***
Fig. 5D	EV vs. STOP1	SMV-GFP positive area ratio (left/right)	<0.0001	***
Fig. 5D	EV vs. GIP2	SMV-GFP positive area ratio (left/right)	<0.0001	***
Fig. 5D	EV vs. HrBP1	SMV-GFP positive area ratio (left/right)	<0.0001	***
Fig. 5D	EV vs. SiPPase	SMV-GFP positive area ratio (left/right)	0.0076	**
Fig. 5F	STOP1 vs. 6K2	SMV-GFP signal intensity	<0.0001	***
Fig. 5F	STOP1 vs. Coexpression	SMV-GFP signal intensity	<0.0001	***
Fig. 5F	GIP2 vs. HC-Pro	SMV-GFP signal intensity	<0.0001	***
Fig. 5F	GIP2 vs. Coexprssion	SMV-GFP signal intensity	<0.0001	***
Fig. 5F	HrBP1 vs. 6K1	SMV-GFP signal intensity	<0.0001	***
Fig. 5F	HrBP1 vs. Coexpression	SMV-GFP signal intensity	<0.0001	***
Fig. 5F	SiPPase vs. NIa-Pro	SMV-GFP signal intensity	0.0011	**
Fig. 5F	SiPPase vs. Coexpression	SMV-GFP signal intensity	<0.0001	***
